# Infantile Hepatic Hemangioendothelioma Associated With Congestive Heart Failure: Two Case Reports With Different Outcomes

**DOI:** 10.1097/MD.0000000000002344

**Published:** 2015-12-31

**Authors:** Tao Wang, Yibin Wang, Yun Liang, Guoyan Lu

**Affiliations:** From the Department of Pediatric Cardiology, Sichuan University West China Second University Hospital, Chengdu (TW, YW, GL); and Department of Pediatrics, Affiliated Hospital of North Sichuan Medical College, Nanchong, Sichuan, China (YL).

## Abstract

Infantile hepatic hemangioendothelioma (IHH) is rare which can regress spontaneously. Arteriovenous shunts within hemangiomas, however, may result in pulmonary artery hypertension (PAH) and congestive heart failure (CHF).

The authors report 2 young infants suffering from multifocal IHH associated with CHF were both treated with glucocorticoid and transcatheter arterial embolization (TAE), but had different outcomes. The PAH decreased immediately and the symptoms of CHF were alleviated after TAE for both of them. For the Tibetan infant, the development was normal with tumor regression by follow-up. For the Han ethnic neonate, PAH increased again in the seventh day with progressive cardiovascular insufficiency. Ultrasound showed a persisting perfusion caused by collateralization around occluded main feeders. Furthermore, a pulmonary infection occurred and ventilation was performed. As a result, the infant died from multiorgan failure caused by CHF and infection.

TAE is a treatment of reducing shunting for hemangiomas. Fistula recanalization in multifocal IHH, however, might be an important risk factor affecting the outcome of TAE. TAE should be further evaluated with special attention to anatomy of feeding and draining vessels, and cardiopulmonary conditions. In addition, the patients were susceptible to secondary pulmonary infection because of lung congestion. As well, the infant from the high altitude area showed better adaptability to hypoxia.

## INTRODUCTION

Infantile hepatic hemangioendothelioma (IHH) is a very rare disease with an incidence of approximately 1 in 20,000 and a male-to-female ratio of 1.3:1–2:1.^1^ Histologically, IHH is a benign vascular tumor and can regress spontaneously without complications. Arteriovenous shunts within hemangiomas associated with hepatic artery-hepatic vein or hepatic artery-portal vein, however, may result in pulmonary artery hypertension (PAH) and congestive heart failure (CHF). And the showing hemodynamic features are similar to the left-to-right shunts in congenital heart diseases. The mortality could reach 90%.^1,2^ Glucocorticoids, interferon-α, and propranolol are commonly prescribed in patients with IHH.^1–3^ When medication is ineffective for the heart failure caused by arteriovenous shunts, transcatheter arterial embolization (TAE) is considered.^3,4^ In the report, we present the experience in 2 patients suffering from multifocal IHH associated with CHF.

### Presenting Concerns

Patient 1 was a female Tibetan infant, 5 months of age, born at full term in Lhasa with a high altitude of 4000 meters, and weighed 3000 g at birth. The mother was a G_4_P_4_ (G=Gravida, P=Para. G4P4: the number of pregnancies is 4, and the number of deliveries is 4) and underwent a vaginal delivery. There were no complications during pregnancy and family history. The infant had symptoms of cough, dyspnea, and cyanosis after crying 3 months before hospitalization. One month ago, these symptoms were aggravated and complicated by oliguria and fever. The infant was diagnosed with severe pulmonary hypertension, cardiac insufficiency, and bronchitis at the People's Hospital of Lhasa. Milrinone and captopril were given to improve cardiac function; Cefotaxime sodium for bronchitis. The patient still, however, had a persistent cough, dyspnea, and fever after discharge.

Patient 2 was a female Han ethnic neonate, 19 days of age, who was evaluated in the emergency department for dyspnea of 2 days duration with aggravated cyanosis for 1 day. The infant was born at full term in Chengdu with an altitude of 500 meters, and weighed 3450 g at birth. The mother was a G_2_P_2_ (G=Gravida, P=Para. G2P2: the number of pregnancies is 2, and the number of deliveries is 2) and underwent a cesarean section. The pregnancy was complicated by polyhydramnios, and there was no family history.

### Clinical Findings

**FIGURE 1:**
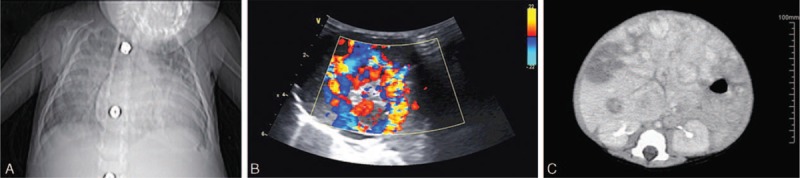
Chest radiograph, Doppler ultrasound, and computed tomography of patient 1. A, chest radiograph showing heart enlargement and a cardiothoracic ratio of 0.75. B, Doppler ultrasound showing a non-uniform echo pattern with prominent vascularity in the left lobe of the liver. C, Abdominal computed tomography with contrast revealed intrahepatic multiple nodules, or circular enhancement with irregular margins, but the central region was non-enhanced. Cystic part or low-density component around the lesion.

The Tibetan infant was hospitalized for coughing and dyspnea with cyanosis of 3 months duration and aggravated oliguria for 1 month. The physical examination showed the following signs: HR 180/min; R 50/min; and BP 79/52 mm Hg. The infant had mild jaundice, and multiple skin hemangiomas of varying sizes. The heart rhythm was normal, with a 2/6 systolic murmur and the second heart sound was enhanced. Abdominal distention was noted. The liver was palpable 4.5 cm below the subcostal margin. The laboratory examinations are summarized in Table 1. A cardiothoracic ratio of 0.65 was measured on the chest radiograph (Fig. 1A). An electrocardiogram indicated biatrial enlargement, biventricular hypertrophy. Echocardiography suggested generalized cardiac enlargement (left ventricle = 30 mm, left atrium = 23 mm, right atrium (RA) = 36 mm, and right ventricle (RV) = 18 mm), moderate pulmonary hypertension (54 mm Hg), and the dilated inferior vena cava with an inner diameter of 15 mm; the ejection fraction and fractional shortening were 72% and 40%, respectively. An abdominal Doppler ultrasound revealed multiple intrahepatic space-occupying lesions and abundant blood flow in tumor (Fig. 1B). An abdominal CT scan showed an enlarged, irregularly shaped liver with multiple intrahepatic masses (Fig. 1C).

**TABLE 1:**
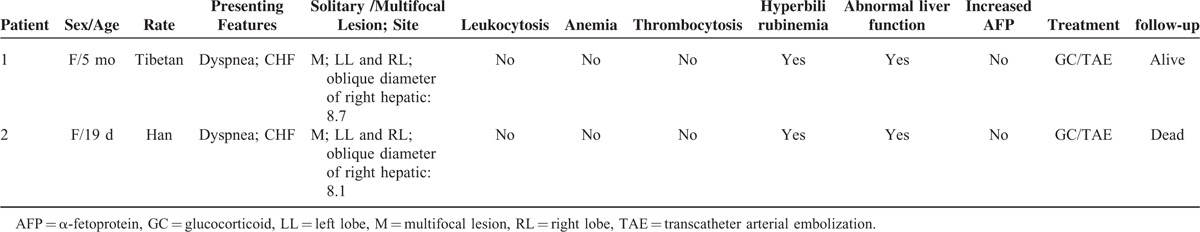
Clinical Presentations

**FIGURE 2:**
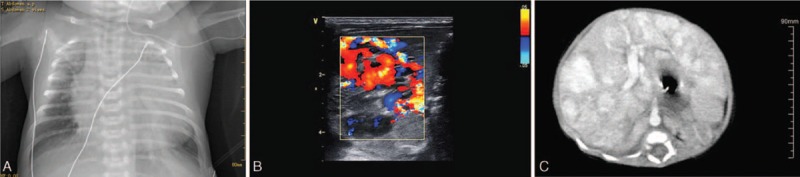
Chest radiograph, Doppler ultrasound. and computed tomography of patient 2. A, Chest radiograph showing heart enlargement and a cardiothoracic ratio of 0.75. B, Doppler ultrasound showing a nonuniform echo pattern with prominent vascularity in the left lobe. C, Abdominal computed tomography with contrast revealed tortuous dilatation of the hepatic artery, intrahepatic portal vein, and hepatic vein, and uniform enhancement of intrahepatic multiple nodules.

The Han ethnic neonate, manifested progressive respiratory distress with cyanosis. The physical examination showed the following signs: HR. 193/min; R. 62/min; and BP. 65/34 mm Hg. The infant had moderate jaundice, and multiple dark red skin hemangiomas of varying sizes. Abdominal distention was obvious. The liver was palpable 3 cm below the subcostal margin. The spleen was palpable 1 cm below the subcostal margin. The laboratory examinations are showed in Table 1. A chest radiograph demonstrated heart enlargement and a cardiothoracic ratio of 0.75 (Fig. 2A). An electrocardiogram showed biventricular hypertrophy. Echocardiography suggested severe pulmonary hypertension (90 mm Hg), severe tricuspid regurgitation, and right atrial enlargement (RA = 20 mm and RV = 35 mm); the ejection fraction and fractional shortening was 64% and 33%, respectively. An abdominal Doppler ultrasound and CT scan confirmed the liver enlargement, multiple space-occupying lesions, an abundant blood supply, and apparent dilatation of the hepatic artery and hepatic vein and inferior vena cava (Fig. 2B and C).

Timeline

**Table:**
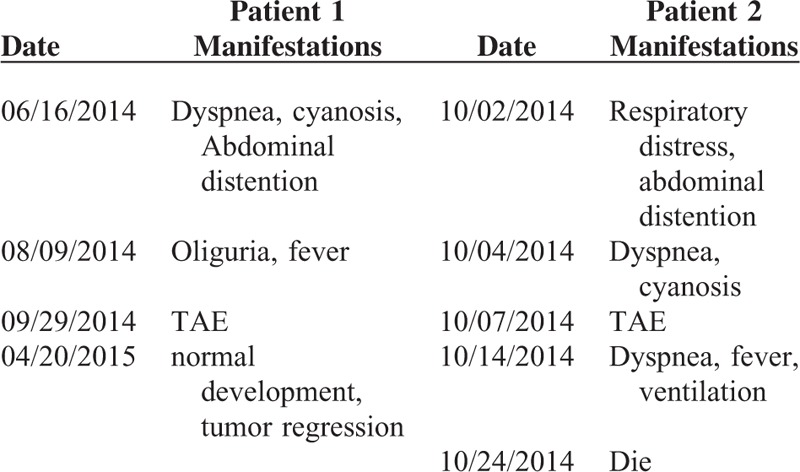

### Diagnostic Focus and Assessment

Infantile hepatic hemangioendothelioma is a benign tumor that presents mostly before the age of 6 months. It is necessary to differentiate it with hepatoblastomas. The diagnosis of the disease mainly depends on imaging and clinical manifestations. Doppler ultrasound is the first choice for the diagnosis of liver tumors, CT can further clarify the diagnosis and determine the extent of the lesion. Doppler ultrasound and CT scan of the 2 infants showed multiple space-occupying lesions, an abundant blood supply, and dilatation of the hepatic artery and hepatic vein and inferior vena cava.

### Therapeutic Focus and Assessment

Oxygen therapy, sedatives, hydrochlorothiazide, spironolactone, digoxinum, and dopamine were prescribed for patient 1. The heart failure symptoms improved greatly. TAE was performed using the Seldinger technique for right femoral artery access, followed by selective catherization with a F4 catheter inserted via the common hepatic artery. An arteriovenous shunt was detected by hepatic angiography. Supraselective embolization of arterial feeding vessels was performed using a 3F occlusion system and flexible coils. The average peak velocity of blood flow in the hepatic artery, as measured by ultrasonography, decreased from 180 cm/s preoperation to 75 cm/s.

For patient 2, oxygen therapy, sedatives, diuretics, digoxinum dopamine, captopril were prescribed as well. Transcatheter arterial embolization was performed using a 3F occlusion system and flexible coils similar to the procedure of patient 1. The average peak velocity of blood flow in the hepatic artery, decreased from 200 cm/s preoperation to 35 cm/s. Pulmonary artery hypertension was reduced to 40 mm Hg from 90 mm Hg. The symptoms, however, were aggravated again, seventh day later. The liver and spleen was further enlarged, and PAH increased again. No relief was achieved with ventilation and digoxinum and dopamine treatment. A pulmonary infection occurred during the course of treatment and meropenem were prescribed. Ultrasound showed a persisting perfusion caused by collateralization around occluded main feeders. As a result, the infant died from multiorgan failure caused by CHF and infection.

### Follow-up and Outcomes

For patient 1, the symptoms of heart failure were alleviated, with a decrease in pulmonary artery pressure to 34 mm Hg from 54 mm Hg. Then the infant was discharged to home after systemic treatment and was prescribed prednisone (2 mg/kg/d). By follow-up at 6 months, the infant was developing normally, and hepatic hemangiomas regressed on ultrasound. But patient 2 died from multiorgan failure causing by uncontrollable infection at the age of 39 days. The 2 young infants suffering from multifocal IHH associated with CHF were both treated with glucocorticoid and TAE, but had different outcomes.

## DISCUSSION

Infantile hepatic hemangioendothelioma is the most common type of infantile hepatic hemangioma, which is usually benign and may be concurrent with vascular malformations of the skin, brain, digestive tract, and other organs. Fatal complications of IHH include high-output CHF, consumption coagulopathy, and Kasabach-Merritt syndrome.^3,5^ Approximately 15% of infants with IHH are complicated by CHF.^1,5^ Deaths caused by CHF account for 70% of all IHH-related deaths.^1,3^ Immediate treatment is crucial for the outcome of the patients. Our understanding of IHH, however, is very limited currently. Treatment options are often based on experience in the treatment of adults or from a small number of infant case reports. There are no detailed and generally accepted standards.

The CHF occured in IHH is associated with arteriovenous shunts in hemangiomas. The arteriovenous shunts can result in a decrease of systemic blood volume and increase of pulmonary blood volume, thus the cardiac output increase. And aggravated by PAH, the fistulas progressively expand with enhanced diversion and finally result in high-output CHF.^4^ The 2 patients described in this study showed extensive arteriovenous shunts and cardiac enlargement (especially the right atrium and right ventricle, and we think these lesion resulted from PAH), heart insufficiency, and dilation of the interior vena cava. All of these symptoms were similar to the characteristic symptoms of left-to-right shunts in congenital heart diseases.

TAE is considered as the first choice and the effective treatment for hepatic arteriovenous shunts. There, however, is little experience in IHH. Warmann et al^6^ performed TAE to block the blood-supplying artery using spring coils for 4 IHH patients complicated by heart failure: 3 patients had significantly improved after TAE for focal lesions; however, 1 patient with multiple lesions showed no improvement, subsequently underwent a liver transplantation. Christison-Lagay^7^ and Kassarjian et al^8^ carried out a retrospective study involving 55 patients with hepatic hemangiomas. They recommended early TAE for those patients with focal or multifocal lesions presenting as shunts or those unresponsive to medication (approximately one-third of the cases). Draper et al^9^ cured 3 patients of diffuse lesions by combining medications with multiple embolization procedures, including 1 patient of multifocal lesions; none of the patients underwent liver transplantation.

Transcatheter arterial embolization for IHH is not recommended as first-line treatment for IHH.^10^ When IHH, however, is complicated by arteriovenous malformations and high-output CHF, TAE by an experienced surgeon may be considered.^4,10^ We performed TAE on the 4 young infants suffering from multifocal IHH. Pulmonary artery hypertension decreased immediately and the symptoms of CHF were alleviated after TAE. Moreover, for patient 1, the development was normal with tumor regression gradually during follow-up. Therefore, supraselective occlusion of feeding vessels represents a controllable and effective procedure.

Transcatheter arterial embolization is considered as a treatment of reducing shunts and counteracting cardiac failure for hepatic hemangiomas. But the efficiency of the interventional approach should be further evaluated with special attention to interindividual variation, anatomy of hemangioma feeding and draining vessels, and cardiopulmonary conditions. The patients with multifocal lesions may undergo TAE followed by medical therapy as a temporary measure while the patient is evaluated for further operation or living transplantation.

The 2 patients, however, had different outcomes. Pulmonary artery hypertension rose again in the seventh day for patient 2. Ultrasound showed a persisting perfusion caused by collateralization around occluded main feeders. Abundant arteriovenous anastomosis in multifocal hemangiomas could raise fistula recanalization. It might be an important risk factor affecting the outcome of TAE in multifocal IHH.

Furthermore, excessive arteriovenous shunts resulted in severe pulmonary congestion, which marked the interstitial fluid increase, and raised the alveolar and airway mucosa edema. This pathophysiological state was beneficial to bacteria and secondary infection. In addition, because of cardiac enlargement and pulmonary artery dilatation, the bronchus and lung tissue were oppressed, and infants were subjected to external pressure resulting in local lung tissue overinflation or atelectasis. The changes of hemodynamics made patients vulnerable to severe pneumonia.

The Tibetan Plateau is one of the highest regions on Earth with an average elevation of 4000 meters. Tibetan highlanders are adapted to a hypoxic environment and possess a suite of distinctive physiological traits.^11^ As well, the infant from the high altitude area showed better adaptability to hypoxia, which was helpful for the patient suffering from hypoxia.

Therefore, the patient 2 died from multiorgan failure caused by combined effect of several risk factors. We should pay more attention to interindividual variability in IHH, and combined measures are necessary. More knowledge needs to be gained on optimal treatment. We hope that sharing our experience will encourage effective therapy in such rare cases.

### Ethics Statement and Patient Consent

The patient provided written permission for publication of this case reports. This study has been approved by the ethics committee of Sichuan University West China Second University Hospital. Each patient who was enrolled in this study has signed the informed consent.
